# Solar light-driven photocatalytic hydrogen evolution over ZnIn_2_S_4_ loaded with transition-metal sulfides

**DOI:** 10.1186/1556-276X-6-290

**Published:** 2011-04-05

**Authors:** Shaohua Shen, Xiaobo Chen, Feng Ren, Coleman X Kronawitter, Samuel S Mao, Liejin Guo

**Affiliations:** 1 State Key Laboratory of Multiphase Flow in Power Engineering, Xi'an Jiaotong University, Xi'an, Shaanxi 710049, China; 2 Lawrence Berkeley National Laboratory, Berkeley, CA 94720, USA

## Abstract

A series of Pt-loaded MS/ZnIn_2_S_4_ (MS = transition-metal sulfide: Ag_2_S, SnS, CoS, CuS, NiS, and MnS) photocatalysts was investigated to show various photocatalytic activities depending on different transition-metal sulfides. Thereinto, CoS, NiS, or MnS-loading lowered down the photocatalytic activity of ZnIn_2_S_4_, while Ag_2_S, SnS, or CuS loading enhanced the photocatalytic activity. After loading 1.0 wt.% CuS together with 1.0 wt.% Pt on ZnIn_2_S_4_, the activity for H_2_ evolution was increased by up to 1.6 times, compared to the ZnIn_2_S_4_ only loaded with 1.0 wt.% Pt. Here, transition-metal sulfides such as CuS, together with Pt, acted as the dual co-catalysts for the improved photocatalytic performance. This study indicated that the application of transition-metal sulfides as effective co-catalysts opened up a new way to design and prepare high-efficiency and low-cost photocatalysts for solar-hydrogen conversion.

## Introduction

Since the discovery of photo-induced water splitting on TiO_2_ electrodes [1], solar-driven photocatalytic hydrogen production from water using a semiconductor catalyst has attracted a tremendous amount of interest [2,3]. To efficiently utilize solar energy, numerous attempts have been made in recent years to realize different visible light-active photocatalysts [4-8]. Among them, sulfides, especially CdS-based photocatalysts with narrow band gaps, proved to be good candidates for photocatalytic hydrogen evolution from water under visible light irradiation [9-12]. However, CdS itself is not stable for water splitting, and Cd^2+^ is hazardous to environment and human health. A number of nontoxic multicomponent sulfides without Cd^2+^ ions have been developed to show comparable photocatalytic efficiency for hydrogen evolution [13-17]. In our previous work [18-22], hydrothermally synthesized ZnIn_2_S_4_ was found to have photocatalytic and photoelectrochemical properties that made it a good candidate for hydrogen production from water under visible light irradiation. On the other hand, a solid co-catalyst, typically noble metal (e.g., Pt, Ru, Rh) [23] or transition-metal oxide (e.g., NiO [24], Rh_2-y_Cr_y_O_3 _[25], RuO_2 _[26], IrO_2_[27]), loaded on the surface of the base photocatalyst can be beneficial to photocatalytic H_2_ and/or O_2_ evolution for water splitting [25]. Nevertheless, there have been only a limited number of studies in which metal sulfides acted as co-catalysts to enhance the activity of a semiconducting photocatalyst [28-30]. For instance, Li and co-workers observed that dual co-catalysts consisting of noble metals (Pt, Pd) and noble-metal sulfides (PdS, Ru_2_S_3_, Rh_2_S_3_) played a crucial role in achieving very high efficiency for hydrogen evolution over CdS photocatalyst [29,30]. In this study, a series of transition-metal sulfides (MS: Ag_2_S, SnS, CoS, CuS, NiS, and MnS) were deposited on hydrothermally synthesized ZnIn_2_S_4_ by a simple precipitation process. The photocatalytic activities for hydrogen evolution over these MS/ZnIn_2_S_4_ products were investigated. We demonstrated that transition-metal sulfides, such as CuS, combined with Pt could act as dual co-catalysts for improving photocatalytic activity for hydrogen evolution from a Na_2_SO_3_/Na_2_S aqueous solution under simulated sunlight.

### Experimental section

All chemicals are of analytical grade and used as received without further purification. ZnIn_2_S_4_ products were prepared by a cetyltrimethylammoniumbromide (CTAB)-assisted hydrothermal synthetic method as described in our previous studies [18,19]. For the synthesis of MS/ZnIn_2_S_4 _(MS = Ag_2_S, SnS, CoS, CuS, NiS, and MnS), 0.1 g of prepared ZnIn_2_S_4 _was dispersed in 20 mL of distilled water and ultrasonicated for 20 min. Under stirring, a desired amount of 0.1 M AgNO_3 _(J.T.Baker Chemical Co., Phillipsburg, NJ, USA), SnCl_2 _(Sigma-Aldrich, Milwaukee, WI, USA), Co(NO_3_)_2_ (Aldrich), Cu(NO_3_)_2 _(Fluka Chemical Company, Buchs, Switzerland), Ni(NO_3_)_2 _(Fluka), or Mn(CH_3_COO)_2 _(Alfa-Aesar, Ward Hill, MA, USA) aqueous solution was dropped into the above suspension, followed by a drop-wise addition of 0.1 M Na_2_S·9H_2_O (Sigma-Aldrich) aqueous solution, containing double excess of S^2- ^relative to the amount of metal ions. The resulting suspension was stirred for another 20 min, then the MS/ZnIn_2_S_4 _precipitate was collected by centrifugation and washed with distilled water for several times, and dried overnight at 65°C. The weight contents of transition-metal sulfides (MS) in these MS/ZnIn_2_S_4_ products were controlled at 0.5% to approximately 2.0%.

X-ray diffraction patterns were obtained from a PANalytical X'pert diffractometer (PANalytical, Almelo, The Netherlands) using Ni-filtered Cu Kα irradiation (wavelength 1.5406 Å). UV-visible absorption spectra were determined with a Varian Cary 50 UV spectrophotometer (Varian Inc, Cary, NC, USA) with MgO as reference. Morphology inspection was performed with a high-resolution scanning electron microscope (SEM, Hitachi S-4300, Tokyo, Japan). Transmission electron microscopy (TEM) study was carried out on a JEOL JEM 2010 instrument (JEOL Ltd., Tokyo, Japan). The X-ray photoelectron spectroscopy (XPS) measurements were conducted on a Kratos spectrometer (AXIS Ultra DLD, Shimadzu/Kratos Analytical, Hadano, Kanagawa, Japan) with monochromatic Al K_α_ radiation (hν = 1,486.69 eV) and with a concentric hemispherical analyzer. Elemental Analysis was conducted on the Bruker S4 PIONEER X-ray fluorescence spectrum (XRF, Bruker AXS GmbH, Karlsruhe, Germany) using Rh target and 4-kW-maximum power.

Photocatalytic hydrogen evolution was performed in a side-window reaction cell. A 300-W solar simulator (AM 1.5; Newport Corporation, Irvine, CA, USA) was used as the light source. The amount of hydrogen evolved was determined using a gas chromatograph (CP-4900 Micro-GC, thermal conductivity detector, Ar carrier; Varian Inc., Palo Alto, CA, USA). In all experiments, 100 mL of deionized water containing 0.05 g of catalyst and 0.25 M Na_2_SO_3_/0.35 M Na_2_S mixed sacrificial agent was added into the reaction cell. Here, sacrificial agent was used to scavenge photo-generated holes. Argon gas was purged through the reaction cell for 30 min before reaction to remove air. Pt (1.0 wt.%) as a co-catalyst for the promotion of hydrogen evolution was deposited in situ on the photocatalyst from the precursor of H_2_PtCl_6_·xH_2_O (Aldrich; 99.9%). In all cases, the reproducibility of the measurements was within ± 10%. Control experiments showed no appreciable H_2_ evolution without solar light irradiation or photocatalyst.

## Results and discussion

The ZnIn_2_S_4_ products prepared by the CTAB-assisted hydrothermal method possessed a hexagonal structure and morphology of microspheres comprising of numerous petals, and showed an absorption edge at about 510 nm (Additional file 1, Figure S1-3). Compared to pure ZnIn_2_S_4_, the obtained MS/ZnIn_2_S_4_ (MS = metal sulfide: Ag_2_S, SnS, CoS, CuS, NiS, and MnS) displayed different absorption profiles (Additional file 1, Figure S4), with enhanced absorption in the visible light region from 550 to 800 nm. Such additional broad band (λ > 550 nm) can be assigned to the absorption of transition-metal sulfides.

**Figure 1:**
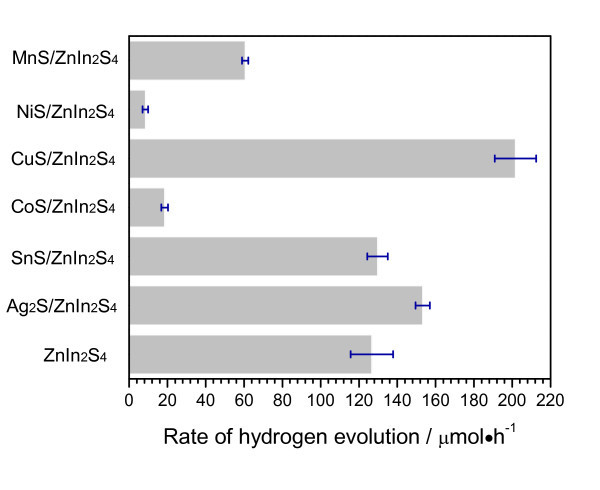
**Average rates of H_2_ evolution**. The average rates of H_2_ evolution over Pt-loaded MS/ZnIn_2_S_4_ (MS = metal sulfide: Ag_2_S, SnS, CoS, CuS, NiS, and MnS) under solar light irradiation in the initial 20-h period.

We investigated the photocatalytic activity for hydrogen evolution over MS/ZnIn_2_S_4_ (MS = metal sulfide: Ag_2_S, SnS, CoS, CuS, NiS, and MnS). Photocatalytic activities for hydrogen evolution over MS/ZnIn_2_S_4_ were evaluated by loading 1 wt.% Pt as co-catalyst. Figure 1 shows the average rates of H_2_ evolution over Pt-loaded MS/ZnIn_2_S_4_ photocatalysts under simulated solar irradiation in the initial 20-h period. The Pt-ZnIn_2_S_4_ showed a photocatalytic activity for H_2_ production at the rate of 126.7 μmol·h^-1^, which is comparable to reported values in previous literatures [18-20]. The hydrogen production rates of Pt-MS/ZnIn_2_S_4_ photocatalysts varied with different kinds of loaded transition-metal sulfides. The Pt-MS/ZnIn_2_S_4_ (MS = Ag_2_S, SnS, and CuS) photocatalysts displayed enhanced activities for hydrogen evolution under solar irradiation. In particular, the H_2_ evolution rate greatly increased to 200 μmol·h^-1^ after loading 1.0 wt.% of CuS on ZnIn_2_S_4_. In this CuS/ZnIn_2_S_4_ sample, the formation of CuS (copper monosulfide) could be evidenced by XPS analysis results shown in Figure S5 (Additional file 1). The survey scan spectrum (Figure S5A of Additional file 1) indicated the presence of Cu, Zn, In, and S in the sample [21,31]. The binding energies shown in Figure S5E (Additional file 1) for Cu 2p_3/2_ and Cu 2p_1/2_ were 952.5 and 932.5 eV, respectively, which are close to the reported value of Cu^2+^[31]. The actual molar ratio of Cu:Zn:In:S was 0.011:0.2:0.39:1.01 as confirmed by XRF analysis result, with weight content of CuS calculated to be 1.15 wt.%, which is quite close to the proposed stoichiometric ratio. The photocatalytic activities for hydrogen evolution over Pt-MS/ZnIn_2_S_4_ (MS = Ag_2_S, SnS, and CuS) in the initial 20-h period were measured to increase in the order of SnS <Ag_2_S <CuS. Generally, these transition-metal sulfides (SnS, Ag_2_S, and CuS) alone are not photocatalytically active for H_2_ evolution, as no H_2_ was detected when they were used as the catalysts. Thus, the improvement of photocatalytic performances of Pt-MS/ZnIn_2_S_4_ (MS = Ag_2_S, SnS, and CuS) can be related to the enhanced separation of photo-generated electrons and holes induced by the hybridization of MS with ZnIn_2_S_4_. In this photocatalysis system, transition-metal sulfides (MS = Ag_2_S, SnS, and CuS) combined with noble-metal Pt acted as dual co-catalysts for photocatalytic hydrogen evolution. However, when transition-metal sulfides (MS = CoS, NiS, and MnS) were loaded on ZnIn_2_S_4_, the rates of H_2_ evolution over Pt-MS/ZnIn_2_S_4_ (MS = CoS, NiS, and MnS) were sharply decreased. Instead of the role as effective co-catalysts, these transition-metal sulfides (i.e., CoS, NiS, and MnS) may work as the recombination center of photo-generated electron-hole pairs, which lowered the photocatalytic activity for hydrogen evolution over Pt-MS/ZnIn_2_S_4_ (MS = CoS, NiS, and MnS). Further investigation is needed and also under way to provide enough supporting information to evidence the negative effects of CoS, NiS, and MnS, although main attention has focused on the more effective co-catalysts such as Ag_2_S, SnS, and CuS in the following discussion.

**Figure 2:**
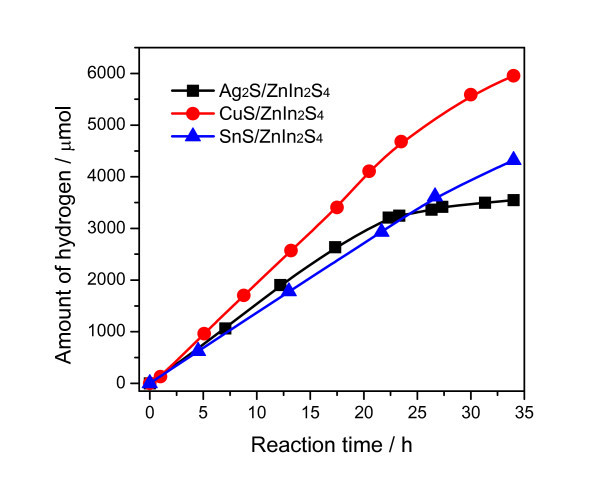
**Time courses of H_2_ evolution**. The time courses of H_2_ evolution over Pt-loaded MS/ZnIn_2_S_4_ (MS = Ag_2_S, SnS, and CuS) under solar light irradiation.

Figure 2 shows the reaction time depended H_2_ evolution over Pt-loaded MS/ZnIn_2_S_4_ (MS = Ag_2_S, SnS, and CuS) under solar irradiation. Pt-SnS/ZnIn_2_S_4_ and Pt-CuS/ZnIn_2_S_4_ exhibited stable activity in the period of 34-h experiment. However, the rate of H_2_ production over Pt-Ag_2_S/ZnIn_2_S_4_ had a significant drop after irradiation for approximately 20 h. This deactivation may result from gradual reduction of Ag_2_S particles loaded on the surface of ZnIn_2_S_4_ to metallic Ag by photo-generated electrons during the reaction. Similar deactivation of photocatalyst was previously observed for CdS modified with Ag_2_S [32]. However, this result is quite different from our previous report on Pt-Ag_2_S/CdS, in which the high dispersion of Ag_2_S in the nanostructure of CdS contributed to stable photocatalytic activity for hydrogen evolution [33]. Taking into account the reduction potential (*vs*. normal hydrogen electrode (NHE)) of Ag^+^/Ag (0.80 V), Cu^2+^/Cu (0.34 V), and Sn^2+^/Sn (-0.14 V), reduction of Ag_2_S by photo-generated electrons is easier than photoreduction of CuS and SnS. Therefore, Pt-MS/ZnIn_2_S_4_ (MS = SnS and CuS) turned to be more stable than Pt-Ag_2_S/ZnIn_2_S_4_ during the photocatalytic reaction for hydrogen evolution.

Table 1 shows the dependence of photocatalytic activity for H_2_ evolution over Pt-loaded MS/ZnIn_2_S_4_ (MS = SnS and CuS) on the loading amount of transition-metal sulfides. With the increase of SnS-loading from 0 to 2.0 wt.%, the rate of H_2_ evolution over Pt-SnS/ZnIn_2_S_4_ does not show significant changes. In contrast, the photocatalytic performance of Pt-CuS/ZnIn_2_S_4_ depends strongly on the amount of CuS-loading, and the optimum loading amount of CuS is approximately1.0 wt.%. The progressive regression of photocatalytic activity observed with the amount of CuS increasing from 1.0 to 2.0 wt.% may be due to the fact that excess CuS particles loaded on the surface of ZnIn_2_S_4_ could act as the optical filter or charge recombination center instead of co-catalyst for charge separation [19,32].

**Table 1:** Average rates of H_2_ evolution over Pt-loaded MS/ZnIn_2_S_4_.

Photocatalyst MS/ZnIn_2_S_4_	Content of MS	Rate of hydrogen evolution μmol/h
ZnIn_2_S_4_	0	126.7
SnS/ZnIn_2_S_4_	0.5%	115.4
SnS/ZnIn_2_S_4_	1.0%	129.7
SnS/ZnIn_2_S_4_	2.0%	127.1
CuS/ZnIn_2_S_4_	0.5%	181.4
CuS/ZnIn_2_S_4_	1.0%	201.7
CuS/ZnIn_2_S_4_	2.0%	139.4

The average rates of H_2_ evolution over Pt-loaded MS/ZnIn_2_S_4_ (MS = metal sulfide: SnS and CuS) under solar light irradiation in the initial 20-h period

**Figure 3:**
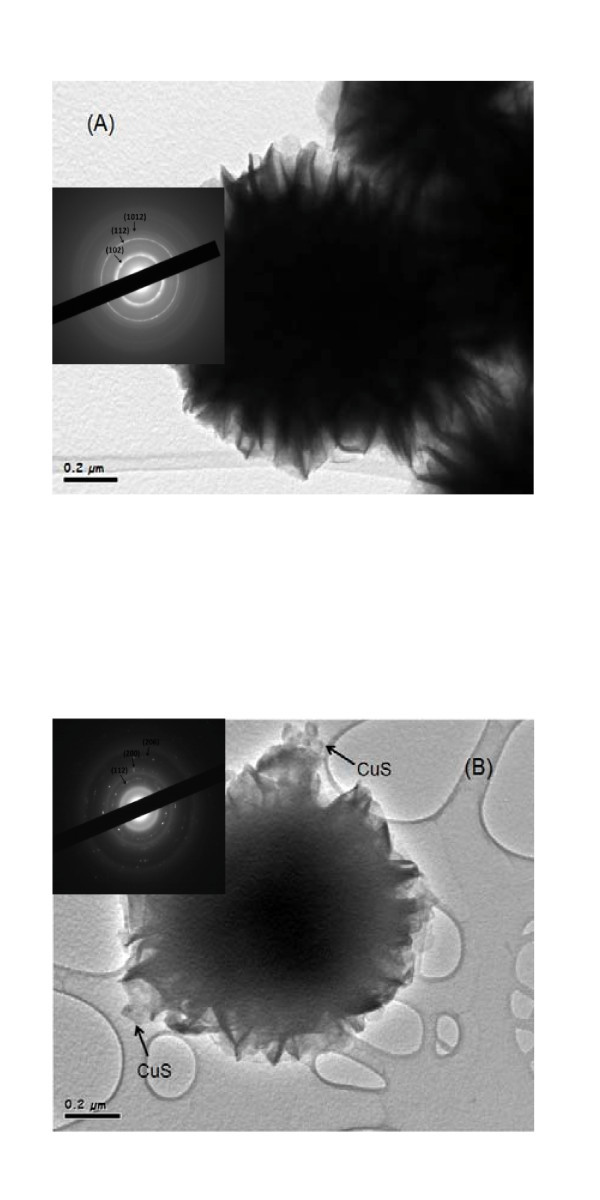
**TEM images (A) ZnIn_2_S_4_ and (B) CuS/ZnIn_2_S_4_**.

To visualize hybridization of CuS with ZnIn_2_S_4_, ZnIn_2_S_4_, and CuS/ZnIn_2_S_4 _photocatalysts were investigated by TEM. A representative TEM image of ZnIn_2_S_4_ is shown in Figure 3A, which shows the formation of microspheres, 1-2 μm in diameter and comprised of a circle of micro-petals. The ED pattern (inset of Figure 3A) substantiates that the ZnIn_2_S_4_ microsphere is of a hexagonal phase. The TEM image in Figure 3B shows that some nanoparticles are loaded on the surface of ZnIn_2_S_4_ microspheres. Such nanoparticles were confirmed by the ED pattern (inset in Figure 3B) to be CuS with typical orthorhombic structure. Thus, nanosized CuS particles dispersed on the ZnIn_2_S_4_ surface would act as the charge-transfer co-catalyst, together with photodeposited Pt particles. The Pt-CuS dual co-catalysts improved the charge separation and therefore increased the photocatalytic activity.

**Figure 4:**
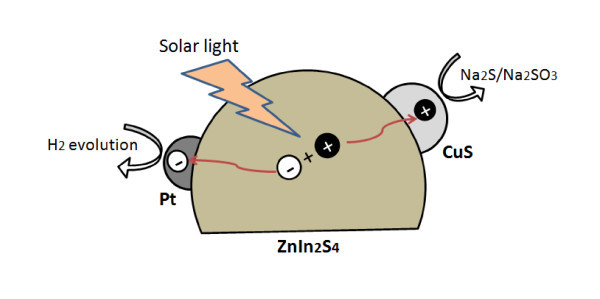
**Schematic illustration of photo-generated charge-transfer process for photocatalytic hydrogen evolution over Pt-CuS/ZnIn_2_S_4_**. From an aqueous solution containing Na_2_SO_3_/Na_2_S under simulated solar light.

Figure 4 illustrates the process of photo-generated charge transfer for photocatalytic hydrogen evolution over Pt-CuS/ZnIn_2_S_4_ in an aqueous solution containing Na_2_SO_3_/Na_2_S under simulated sunlight. Band gap excitation produces electron-hole pairs in ZnIn_2_S_4_ particles. The excited electrons are subsequently channeled to Pt sites, which reduce protons to generate hydrogen. On the other hand, the valence band potential of ZnIn_2_S_4_, deduced from the conduction band potential (0.29 V *vs*. NHE) [22] and the band gap energy (2.43 eV), is about 2.72 V *vs*. NHE, which is more positive than the OH^-^/O_2_ redox potential [4]. The valence band potential of CuS is less positive than the OH^-^/O_2_ redox potential [34]. Such a difference of valence band potentials makes it possible for the excited holes to transfer from ZnIn_2_S_4_ to CuS to react with Na_2_S/Na_2_SO_3_ electron donor in the solution. Therefore, Pt and CuS are supposed to act as the reduction and oxidation co-catalyst, respectively, which leads to more efficient charge separation, thus improves photocatalytic activity of Pt-CuS/ZnIn_2_S_4_. Similar benefits of dual co-catalysts on photocatalytic activity have been observed for CdS loaded with noble metals as reduction catalysts and noble-metal sulfides as oxidation catalysts [29,30]. It is noteworthy that replacing noble-metal sulfides (such as PdS) by transition-metal sulfides (such as CuS) as the co-catalysts would help lower the cost of photocatalysts for solar-hydrogen production. Moreover, seeking effective co-catalyst candidates could be expanded to other transition-metal sulfides such as FeS and SnS_2_, etc. Detailed research on this subject is still an ongoing progress in our group.

## Conclusions

In summary, a series of Pt-loaded MS/ZnIn_2_S_4_ (MS = transition-metal sulfides: Ag_2_S, SnS, CoS, CuS, NiS, and MnS) photocatalysts were developed. It is found that Ag_2_S, SnS, and CuS could enhance the photocatalytic activity of hydrogen evolution over ZnIn_2_S_4_ to varying degrees, while SnS, CoS, and NiS would reduce the activity. Among them, the Pt-CuS/ZnIn_2_S_4_ photocatalyst exhibited the most efficient and stable activity for hydrogen evolution. This can be attributed to the fact that the dual co-catalysts of Pt and CuS entrapped photo-induced electrons and holes for reduction and oxidation reaction, respectively, improving charge separation and hence the photocatalytic activity. Application of transition-metal sulfides as co-catalysts opens up an opportunity toward realizing high-efficiency, low-cost photocatalysts for solar-hydrogen conversion.

## Competing interests

The authors declare that they have no competing interests.

## Authors' contributions

SS carried out experiments except SEM and TEM characterization, and drafted the manuscript. XC participated in the design of the study. FR performed the TEM characterization. CXK performed the SEM characterization and improved English writing. SSM provide financial support and participated in the design and coordination of this study. LG conceived of the study, and participated in its design and coordination. All authors read and approved the final manuscript.

## Supplementary Material

Additional file 1 **Figures S1, S2, S3, S4 and S5**. Click here for file
