# Supplementary material for: Solar light-driven photocatalytic hydrogen evolution over ZnIn2S4 loaded with transition-metal sulfides
Source: Nanoscale Res Lett. 2011 Apr 5;6(1):290. doi: 10.1186/1556-276X-6-290 (PMC3211356; doi:10.1186/1556-276X-6-290)
Supplement: Additional file 1 — Figures S1, S2, S3, S4 and S5. [file 1556-276X-6-290-S1.DOC]

**Electronic supplementary material**

Solar-Light-Driven Photocatalytic Hydrogen Evolution over ZnIn_2_S_4_ Loaded with Transition-Metal Sulfides

Shaohua Shen^1,2^ · Xiaobo Chen^2^ · Feng Ren^2^ · Coleman X. Kronawitter^2^ · Samuel S. Mao^2^ · Liejin Guo[[1]](#footnote-2)

Figure S1. XRD pattern of ZnIn_2_S_4_ prepared by CTAB-assisted hydrothermal method.


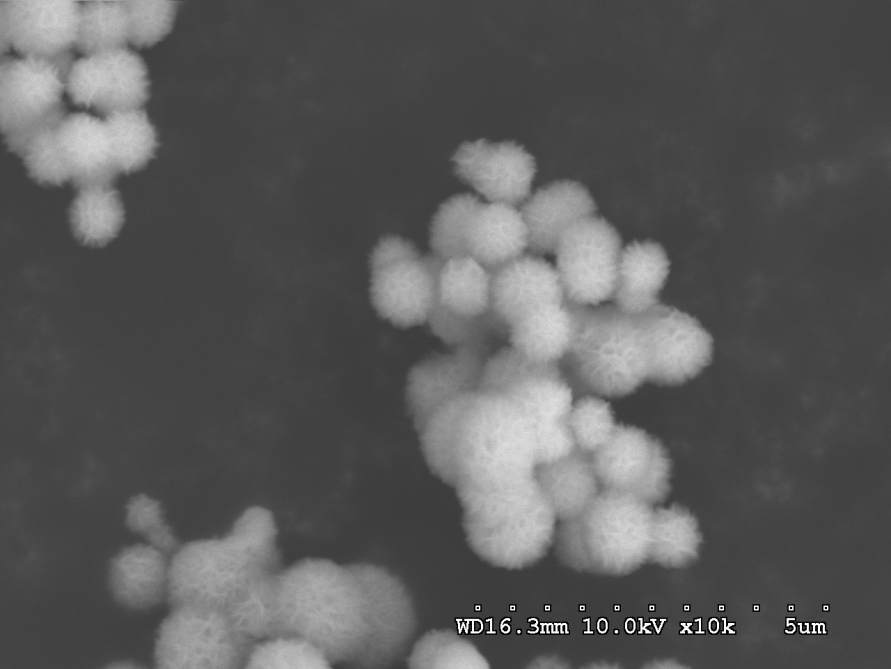

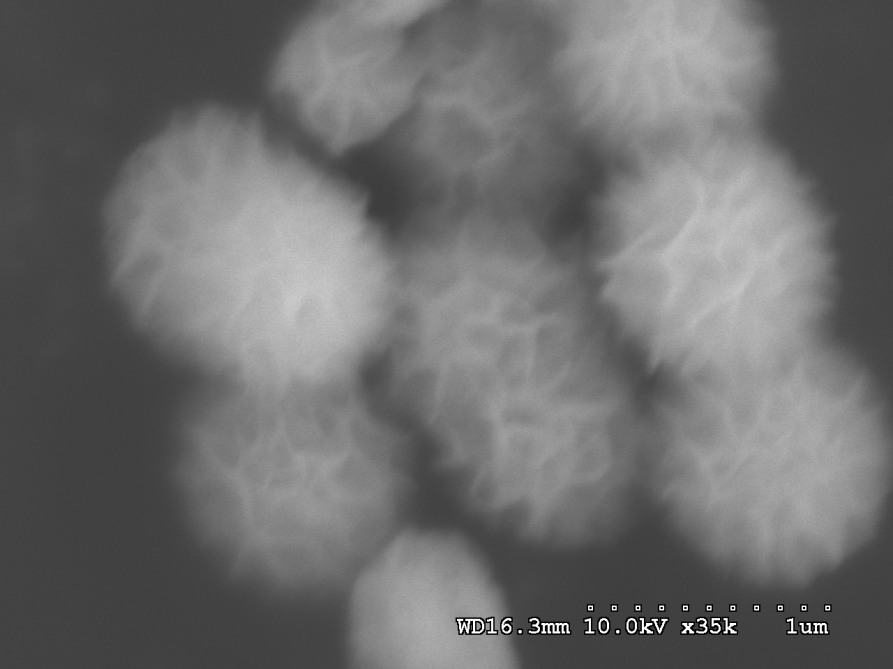


Figure S2. SEM images of ZnIn_2_S_4_ prepared by CTAB-assisted hydrothermal method.

Figure S3. UV-Vis diffuse reflection spectra of ZnIn_2_S_4_ prepared by CTAB-assisted hydrothermal method.

Figure S4. UV-Vis diffuse reflection spectra of MS/ZnIn_2_S_4_ (MS = metal sulfide: Ag_2_S, SnS, CoS, CuS, NiS, and MnS).

Figure S5. XPS analysis of CuS/ZnIn_2_S_4_: (A) survey scan, (B) Zn region, (C) In region, (D) S region and (E) Cu region.

1. State Key Laboratory of Multiphase Flow in Power Engineering, Xi’an Jiaotong University, Xi’an, Shaanxi 710049, China. Email: [lj-guo@mail.xjtu.edu.cn](mailto:lj-guo@mail.xjtu.edu.cn)

   ^2^ Lawrence Berkeley National Laboratory, Berkeley, CA94720, USA. Email: [ssmao@lbl.gov](mailto:ssmao@lbl.gov) [↑](#footnote-ref-2)
